# A Scoping Review on Access and Use of Technology in Youth Experiencing Homelessness: Implications for Healthcare

**DOI:** 10.3389/fdgth.2021.782145

**Published:** 2021-11-24

**Authors:** Shalini Lal, Amané Halicki-Asakawa, Amélie Fauvelle

**Affiliations:** ^1^School of Rehabilitation, University of Montreal, Montreal, QC, Canada; ^2^Youth Mental Health and Technology Lab, Innovation and Evaluation Hub, University of Montreal Hospital Research Centre (CRCHUM), Montreal, QC, Canada; ^3^Douglas Mental Health University Institute, Montreal, QC, Canada

**Keywords:** digital equity, telemedicine, telehealth, cellular phone, internet, eMental health, digital health, mhealth

## Abstract

**Introduction:** Youth are among the fastest growing subset of the homeless population. Youth experiencing homelessness (YEH) face multiple barriers in accessing health information and health care services. As such, they may best be reached through information and communication technologies (ICTs); however, limited efforts have been made to synthesize literature on this topic. In this paper, we review studies on access and use of ICTs among YEH. We also discuss the implications of the review for healthcare.

**Methods:** Using scoping review methodology, we searched four databases (Medline, Embase, PsycInfo, and CINAHL) for studies published between 2005 and 2019, screening 1,927 titles and abstracts.

**Results:** We identified 19 articles reporting on studies with YEH between the ages of 12-30, the majority of which were published in the USA. On average, more than half of the samples owned smartphones, used social media, and accessed the internet weekly to search for housing, employment, health information, and to communicate with family, peers, and health workers; however, many youths faced barriers to sustaining their access to technology. Benefits of using ICTs were connecting with home-based peers, family, and case workers, which was associated with a reduction in substance use, risky sexual health behaviors, and severity of mental health symptoms. Connecting with negative, street-based social ties was identified as the most common risk factor to using ICTs due to its association with engaging in risky sex behaviors and substance abuse.

**Discussion:** This review supports the advancement of research and practice on using ICTs to deliver public health information and health services to YEH, while also considering the health-related risks, benefits, and barriers that YEH face when accessing ICTs.

## Introduction

Youth homelessness is a serious and complex public health issue. Factors leading to situations of youth homelessness are multifaceted, and involve the interaction of issues such as a lack of affordable housing, economic insecurity, behavioral health, violence at home, lack of positive social supports, and involvement in the child welfare system (1, 2). Various definitions have been proposed to describe homelessness. Gaetz et al. (3) define youth experiencing homelessness (YEH) as adolescents and young adults living independently from their caregivers, in unstable or inappropriate housing situations, and lacking the social and material means to successfully transition into adulthood. This definition encompasses youth living on the street, but also the hidden homeless; for example, young people living in hotels and motels, staying with friends, or sleeping in unsafe places, such as cars, tents, or in parks (4–6).

Given the transient nature of the homeless population and heterogeneity in definitions (7, 8), it is challenging to provide accurate estimates of the actual number of YEH (3, 9). Morton et al. (8) found that in the United States of America (USA), between 700,000 and 3.5 million young adults aged 18-25 experience homelessness each year, with Black youth having a significantly higher risk of homelessness. In Canada, approximately 20 percent (or 30,000-40,000 annually) of individuals experiencing homelessness are young adults aged 16-24, with a similar overrepresentation of youth from marginalized communities (i.e., youth identifying as LGBTQIA2SP+, racialized youth) (2, 9–12). In Canada and the USA, reports show that the rates of homelessness in children and adolescents are outpacing other age groups of the homeless population (12, 13).

The health of YEH is of critical concern. Without a stable and safe place to live, they often need to engage in risky activities (e.g., sex trade, selling drugs, carrying a weapon) for basic survival, which may place them at higher risk for developing health problems (12, 14–16). Health issues affecting YEH include, for example, respiratory and dermatology conditions; mood, anxiety, and behavioral disorders; psychosis; attempted suicide; and, substance abuse (11, 12, 14–19). Despite the prevalence of health and social issues among YEH, they are particularly marginalized from the health care system, facing multiple barriers to accessing timely and effective care (12, 16, 18, 20–23). Barriers they face in accessing care include: limited money, difficulties having stable contact information/address/ID, limited knowledge about health services, and negative attitudes and perceptions of healthcare professionals toward the homeless population (12, 16, 20, 22). Consequently, the mortality rates of this population are increased by up to 30 times in comparison to the general public (24–30). Such evidence provides support for the importance of creating interventions and services that are accessible and effective for this population.

Technology-enabled interventions are a promising avenue to address some of the aforementioned barriers and to help improve access to health services for YEH. However, prior to developing and delivering health information and services through technology, it is important to know the extent to which YEH use information and communication technologies (ICTs). A previous review of studies published until 2012 concluded that many homeless persons use ICTs and that there is potential for developing technology-delivered interventions aimed at improving health services among this population (31). However, this review included only a few articles that were focused on a younger population (given the nascence of the research at that time) and considering the evolution in technology development and access, the results of such a review warrant updating. Over the past decade, more studies have focused on examining access and use of ICTs among YEH (32), however limited efforts have been made to synthesize this literature. Such knowledge can be useful for informing public health practice (e.g., communicating knowledge to the homeless youth population during public health emergency situations, such as COVID-19) and health care services more broadly.

As such, we conducted a scoping review with the objective of synthesizing knowledge on access and use of ICTs among YEH and to discuss implications for public health care. Our main research questions were: (1) What is known about the rates of access and use of ICTs among YEH?; (2) what are the factors affecting access and use of ICTs among YEH; (3) why do YEH use ICTs (i.e., for what purposes); (4) what are the health-related benefits and risks for YEH in using ICTs; and, (5) what implications does the existing research have for future health care research and practice?

The scoping review method was chosen as it provides a systematic, rigorous and transparent approach for mapping a field of interest in terms of the volume, nature and characteristics of existing research (33–35). Scoping review methodology has been increasingly used in the health literature (34, 35) and is particularly relevant when reviewers are interested in questions extending beyond intervention effects or in emerging fields of research (33, 36). Given that the study of access and use of ICTs among YEH is a relatively new area of research, a scoping review is an important first step in informing future research and practice.

## Method

Our review is based on Arksey and O'Malley's (33) five-stage framework for conducting scoping reviews and informed by the Preferred Reporting Items for Systematic reviews and Meta-Analyses extension for Scoping Review (PRISMA-ScR) (36). We first developed a scoping review protocol including a rationale for conducting the review, the main objectives, search strategy, inclusion and exclusion criteria, and methods for screening and data extraction, which was then piloted and discussed by the research team before finalizing. The final protocol was registered retrospectively in Open Science Framework (https://osf.io) (protocol registration accessible via: https://doi.org/10.17605/OSF.IO/6NY9B).

### Study Identification and Selection

#### Information Sources and Search Strategy

**Figure 1:**
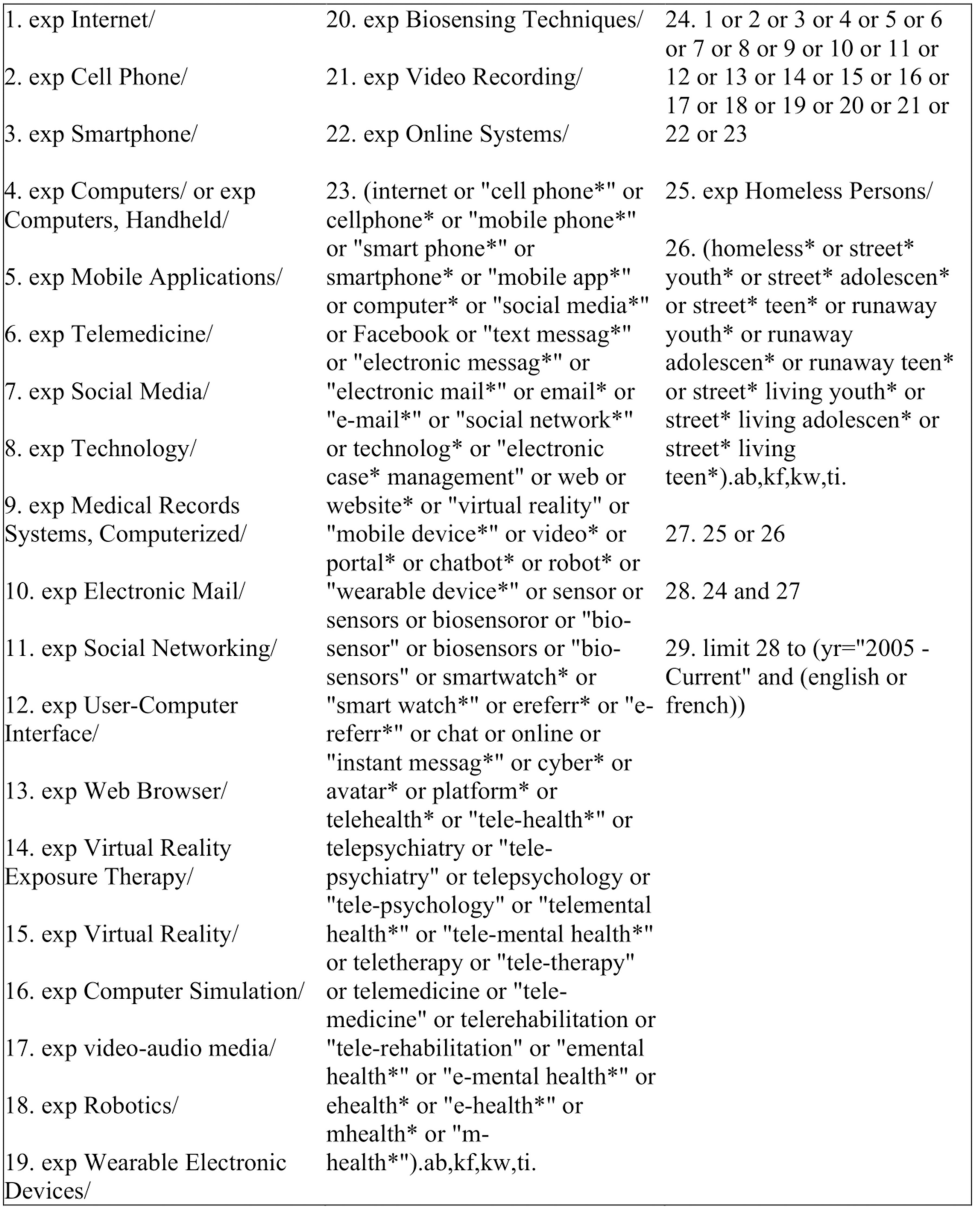
2019 search strategy launched in Medline. * refers to the inclusion of all forms of the word (e.g., plural form).

A literature database search by subject, title, and abstract was applied using Medline, Embase, PsycInfo, and CINAHL. Three consultations were made with a university-based, paramedical librarian to develop a Medline search strategy, as described in Figure 1, which was then adapted for the other databases. The reference lists of selected articles were also screened to obtain additional articles. An initial search strategy was developed and implemented November 2nd, 2015. Given that we did not find a large number of papers to justify a full-review, we conducted a second search on April 19th, 2016 (including revisions to our keyword strategy), and an updated search on March 6th 2019, each time in consultation with the librarian. All searches involved articles published from 2005 in English and French. This date of publication was chosen given that ICTs have been evolving rapidly over the past decade; thus, literature older than 15 years would not be as pertinent to the current landscape of research and practice in this field. No other limitations were placed.

#### Selecting Sources of Evidence

Once the publications were retrieved and duplicates removed using Endnote, the titles and abstracts were screened (the full text was also screened for any articles identified as meeting or potentially meeting the inclusion criteria) to identify documents to be retained for the review based on the following criteria:

##### Inclusion criteria

(1) The publication reports on a study regarding accessibility of ICTs for YEH and/or use of ICTs by YEH; (2) the technology discussed in the document is an ICT (e.g., cell phone, social media, email, electronic case management); (3) the publication is written in English; (4) all types of study designs are included (e.g., qualitative, quantitative, mixed methods, descriptive); and (5) the date of the publication is from 2005 onwards.

##### Exclusion criteria

Publications were excluded based on the following: (1) the publication reports on a technology that is not included in the definition of ICTs used in this review (e.g., medical technology, diagnosis tools); and (2) the publication focuses on the use of an ICT that is only accessible by a healthcare professional (e.g., electronic medical record).

The inclusion and exclusion criteria were piloted on 10% of the documents to ensure their clarity and the ability of the research team to identify relevant articles. Revisions to the criteria were then made and applied to the rest of the retrieved titles and abstracts. A2 and A3 each screened a subset of the titles and abstracts, with any unclear articles reviewed at the full text level and discussed with A1, following which a final decision was made regarding study inclusion.

### Charting the Data and Reporting Results

The selected publications were read, annotated, and entered into a Microsoft Excel spreadsheet. The data extraction sheet was piloted by A3 with two of the included studies, and then revised in consultation with A1. Next, the following information was extracted and classified by two members of the research team (A2, A3): access to technology (including methods of access), use of technology (i.e., frequency of use and purpose), impact on health outcomes, and key conclusions and implications for future research and/or practice. We also extracted basic information, including: authors, publication year, country of publication, study objectives, study design, methods, sample size, and sociodemographic characteristics.

The data extraction of A3 was validated by A2, and the data extraction of A2 was validated by an additional member of the research team. Publications reporting on data from the same study sample were considered a set, and counted as one study in the PRISMA diagram. In terms of summarizing the data, where applicable, simple weighted averages were calculated by A2 in consultation with a statistician based on study sample size for data pertaining to rates of access and use of ICTs, and for sociodemographic information (i.e., studies that did not report on a category of information were not included in the weighted average calculations). The qualitative data (e.g., reasons for using ICTs, methods of access, risks and benefits to ICT use) was coded by A2 and managed using Microsoft Excel and validated by an additional member of the research team. We did not conduct a critical appraisal of the included studies given that this is not typically an objective of conducting a scoping review (33, 35, 36) and the large research design heterogeneity of the studies reviewed.

## Results

**Figure 2:**
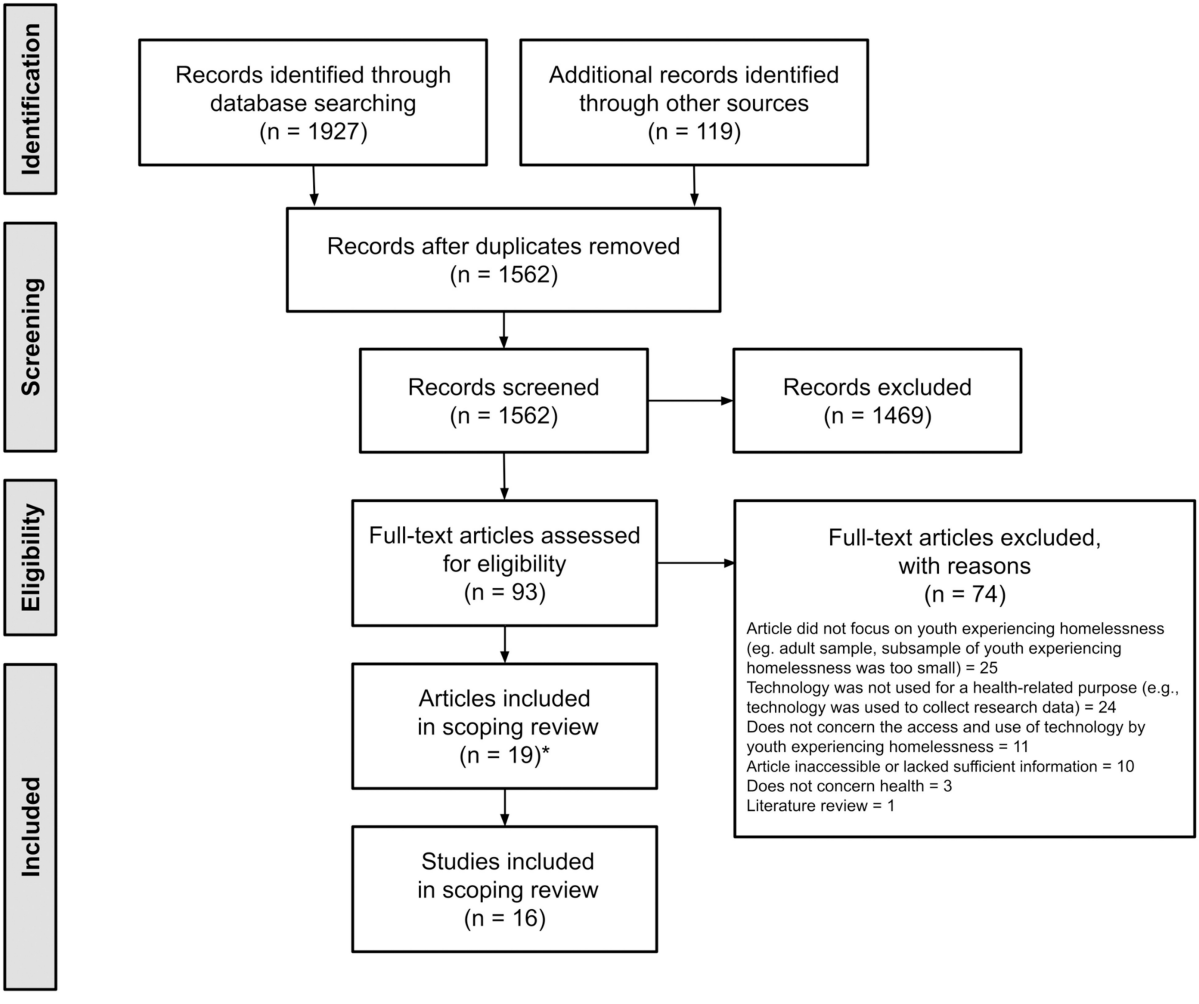
Preferred Reporting Items for Systematic reviews and Meta-Analyses (PRISMA) diagram. *Six of the articles were grouped together into three pairs, as each set reported on data collected from the same sample.

We identified 19 relevant peer-reviewed articles reporting on access and use of ICTs in YEH, though six of these were paired together and considered one set as they reported on data from the same study sample, resulting in a dataset of 16 study samples (see Figure 2 for the adapted PRISMA flow diagram and details on numbers of items screened and excluded, including reasons for exclusions). The 19 articles were published between 2010 and 2018, with 17 from the USA, one from Canada, and one from Australia. Appendix Table 1 (Supplementary Material) provides a summary of the objectives and results of each of the studies included in the review. The total sample was comprised of 3,123 participants (sample sizes ranged from 20 to 829; the majority under 200), aged between 12 and 30 years old, 2,856 (91.5%) of which were YEH living in a variety of housing situations (e.g., shelters, living on the street, temporary housing, etc.). The YEH group comprised of 1,876 (65.9%) males, 898 (31.5%) females, and 44 (1.5%) transgendered individuals, within the 18 papers in which sex was reported. Within the 16 papers that reported on ethnicity, the majority of YEH (*n* = 916; 32.6%) were Black/African American, approximately a third were White (*n* = 859; 30.6%), and the rest were Hispanic/Latinx (*n* = 422; 15%), or mixed race (*n* = 367; 13.1%). See Table 1 for additional sociodemographic information.

**Table 1:** Participant sociodemographics.

	***N***	**%**
Total sample	3,123	100.0%
YEH	2,856	91.5%
YEH sample from studies that examined ethnicity	2,808	98.3%
Black/African American	916	32.6%
White	859	30.6%
Hispanic/Latinx	422	15.0%
Mixed race	367	13.1%
Other	345	12.3%
Unknown/missing information	2	0.1%
YEH sample from studies that examined sex	2,847	99.7%
Male	1,876	65.9%
Female	898	31.5%
Transgender	44	1.5%
Unknown/missing information	29	1.0%
YEH sample from studies that examined sexuality	2,189	76.6%
Heterosexual	1,628	74.4%
LGBTQIA2SP+	558	25.5%
Unknown/missing information	3	0.1%

*YEH, Youth experiencing homelessness*.

### Rates of Access and Use

Four studies reported on the rates that YEH access and use mobile phones; on average, 62.6% owned a mobile phone (32, 37–39). Based on two studies, an average of 68.8% specifically owned a smartphone (32, 38). The range of mobile phone ownership was wide (46.7-100%); for example, one study found that 78% of those in their quantitative analysis (*n* = 41) and 90% of those in their qualitative analysis (*n* = 52) owned a mobile phone (38), whereas another study found that their entire sample (*n* = 22) owned a mobile phone (37).

Eight studies reported on the rates that YEH access and use the internet; on average, 38.2% used the internet at least once daily, with a range of 28-86.5% (32, 40–46). Based on four studies, an average of 55.1% of YEH accessed the internet at least once a week, with a range of 45-93% (41, 43, 44, 46). Two of the studies reported that a significant majority of their sample used the internet regularly, but did not report on the actual frequency of access (32, 42).

Finally, 13 studies found that YEH access and use social media in some capacity (32, 37, 40, 43–52). Based on seven studies, an average of 77.1% used social media, with a range of 57-90.7% (32, 45–49, 52). Two of the studies reported on the frequency of YEH's social media use, with an average of 36.0% reporting daily social media use, and 26.6% reporting weekly use (47, 52).

### How Youth Experiencing Homelessness Access ICTs

Ten studies described how YEH accessed ICTs (32, 38–40, 42–46, 49). Cell phones were sometimes obtained as a gift, purchased with personal money, or with money obtained from panhandling, a job, or federal benefits (32, 38, 39). The internet was commonly accessed via a friend/family member's or publicly-available device; through public libraries and Wi-Fi; and through youth service community agencies, Internet cafes, schools, and workplaces (42–46, 49).

### Reasons for Using ICTs

Eighteen studies addressed the reasons for YEH's use of ICTs (32, 37–53). Reasons for use most frequently cited were to navigate social networking sites (*n* = 13), to communicate with peers (*n* = 11) and family members (*n* = 9), to conduct job-related activities (*n* = 9), for communication generally (e.g., checking email; *n* = 8), and to seek health services (*n* = 5) and health-related information (*n* = 5). Table 2 provides additional details on the reasons for which YEH used ICTs.

**Table 2:** YEH's reasons for using ICTs.

**Reason for using ICTs**	**No of Studies**
Navigating social networking sites (e.g., Facebook, Twitter, MySpace, etc.) (32, 37, 42–52)	13
Communicating with peers (e.g., through email, social media, instant messaging, text message, etc.) (39, 40, 42–44, 47–52)	11
Communicating with family members (e.g., through email, social media, instant messaging, text message, etc.) (39, 40, 42–44, 47–49, 51)	9
Job related activities (e.g., job searching, resume building, etc.) (37, 41–43, 45, 46, 48, 49, 51)	9
General communication/checking email (person they are communicating with not specified) (37–39, 42, 43, 45, 46, 49)	8
Seeking health services (e.g., searching for a doctor or health clinic) (40, 41, 45, 46, 53)	5
Seeking general health-related information (e.g., looking up mental health concerns and symptoms) (40, 41, 45, 46, 53)	5
Entertainment- and leisure-related activities (e.g., listening to music, playing games, watching movies, etc.) (37, 42, 43, 46, 49)	5
Education-related activities (e.g., navigating a school's website or online portal, homework) (37, 43, 46, 48)	4
Finding a place to stay (e.g., searching for apartment listings, shelters, etc.) (41, 42, 46, 49)	4
Dating/relationships (e.g., seeking a sexual partner online, navigating a dating site, etc.) (44, 47, 48, 52)	4
Communicating with case workers (e.g., through email, social media, instant messaging, text message, etc.) (39, 47, 49, 51)	4
Seeking sexual health-related information (e.g., information about HIV prevention) (40, 45, 46)	3
Seeking general information (e.g., using Google) (37, 42)	2
Practical uses (e.g., using a phone as an alarm clock or for navigation) (37)	1

*YEH, Youth experiencing homelessness; ICT, Internet communication technology; HIV, Human immunodeficiency virus*.

### Factors Affecting Access to ICTs

Eleven studies identified factors that affected YEH's access to and use of ICTs (32, 37–42, 45, 46, 49, 54). The most cited factor was the actual living situation of the youth (*n* = 6), with youth experiencing homelessness or street-based living situations reporting less access to ICTs than youth who were able to find housing more consistently (32, 39, 41, 45, 46, 49). For example, one study found that participants residing in a house or an apartment were more likely to engage in regular use of social media (90.6%) than those living on the streets (55.6%) (32). Compared to when they were housed, YEH's internet behaviors became more goal-oriented, with less time spent on leisurely activities or entertainment (46).

Other factors affecting ICT access included the youth's financial situation and the availability of public devices. Unstable financial situations often led to phone deactivation due to missed payments, to sharing devices with a friend, to having the phone stolen, and difficulties in maintaining the device's functionality (e.g., charging the phone) (37, 38, 54). Some participants reported challenges accessing ICTs through public institutions, such as specific hours of operation, long wait times, downloading or printing difficulties, and website restrictions (42).

Despite the barriers they faced in accessing technology, one study found that youth reported comfort in using ICTs (70% self-assessed their computer abilities as better than average, and 85% reported being able to use a computer), due to previous family and school experiences. In addition, youth used ICTs for a diverse range of activities, suggesting a relatively high level of digital literacy (42).

### Risks and Benefits of Using ICTs With Youth Experiencing Homelessness

Eleven studies established a link between the use of ICTs by YEH and to certain risks and benefits (38–40, 42, 44, 47, 49–53). The most common benefit was the ability to connect with positive social ties, such as home-based peers, family members and case workers, which was associated with a reduction in substance use, risky sexual health behaviors, and severity of mental health symptoms (40, 44, 47, 50, 52).

However, connecting with negative, street-based social ties was identified as the most common risk factor to using ICTs due to its association with an increased likelihood of engaging in risky sex behaviors (e.g., exchange sex, sex with someone met online) and substance abuse (40, 44, 47, 52). Further, discussing drinking, drugs, and sex on social networks with street-based ties was linked to an increase in risky health behaviors, in comparison to discussions of love or goals/future plans (47, 52).

## Discussion

### Key Findings in Relation to Access and Use of ICTs

The aims of our scoping review were to examine the ways that YEH access and use ICTs (i.e., frequency of use, purpose of use, barriers faced), and to discuss the implications of the findings for health care. We identified 16 studies (19 articles) demonstrating that there is a growing pool of evidence on access and use of ICTs among YEH, and that the use of ICTs plays an important role in their lives. At the same time, 16 studies of varying research design and sample sizes obtained through methods subject to sampling bias indicates an ongoing need for research on a highly marginalized population in urgent need for health care services (31).

In terms of our research questions, our key findings are: first, studies report high percentages of access to and use of ICTs by YEH (i.e., on average, across studies, 62.6% owned a mobile phone, with 68.8% owning a smartphone; 38.2% accessed the internet daily, with 55.1% reporting weekly access; and 77.1% used social media platforms). In comparison, surveys conducted with housed youth aged 13-17 in the USA and with youth aged 15-24 in Canada found that 92-96% of their samples went online daily, with nearly 75% of youth in the American sample reporting smartphone access (55, 56). The higher rates of ICT access in housed youth are unsurprising, considering that homelessness was linked to a decrease in internet use and access (32, 39, 41, 45, 46). However, it is important to note that the studies we reviewed are subject to sampling bias (i.e., recruitment from shelters, drop ins), and thus should be interpreted with caution. Moreover, accessibility to ICTs may differ depending on the country (e.g., prices of technology, public resources, governmental programs, etc.) and across regional areas (e.g., provinces, states, cities).

YEH diverge from youth in the general population in the methods and barriers to accessing ICTs. Many YEH rely on public computers in libraries and community agencies to access the Internet, which is accompanied by a diverse range of obstacles (e.g., wait lists, restrictions on site searches). Owning a cell phone also represents a financial burden for many YEH, who may need to panhandle or share the device with a friend to afford it, which can lead to its deactivation (38, 39, 43). These barriers can create a discontinuity in the sustainment of various social contacts for YEH and in the implementation of ICTs-based intervention.

Second, in the one study that discussed technology literacy, YEH reported confidence with their ICTs skills, due in part to exposure to technology at a young age (42). We also found that YEH used ICTs for an array of purposes, suggesting that they may be comfortable navigating and engaging with ICTs-enabled health interventions. However, given that few studies have addressed technology literacy in this population, and that research with other young populations shows that youth encounter several challenges in searching the internet for health-related information (57), this topic warrants further attention.

Third, our findings show that technology supported the maintenance of positive and healthy social contacts, which was associated with less depressive symptoms, a reduction in substance-using behaviors and more adequate sexual health behaviors (40, 44, 50, 51). This suggests that high accessibility to ICTs could also allow YEH to maintain good social relationships, influencing health outcomes. However, it should be noted that using ICTs to connect with street-based peers and to discuss drinking, drugs, and sex increased the likelihood of engaging in these risky health behaviors (44, 47, 51, 52). Thus, it is important to be cognizant of the nature of YEH's online connections, and encourage the use of ICTs to maintain positive social contacts.

### Implications for Practice

The use of technology-enabled interventions with homeless populations is a new area of research and practice for healthcare professionals with several elements to consider, including: increasing access to technology, optimizing technology-based infrastructure, providing training for community outreach and health workers, and engaging service users in the development of diverse and contextually-sensitive interventions (58).

The high rates at which YEH are accessing and using ICTs for various goal-oriented behaviors indicates that technology plays a critical role in their lives. Prioritizing free and accessible technology in public settings (e.g., shelters, community centers, libraries, harm reduction centers) and free access to mobile devices, may be an important way to empower YEH, enable them to maintain connections with pro-social peers and family, and help build their awareness of public health guidelines, health services, and information.

Our findings support the notion that ICTs can improve communication with YEH for outreach purposes (31). Considering that YEH access and use a variety of ICTs, health care providers may consider ICT-based forms of communication to provide services and information. Concurrently, diversity in communicating with and disseminating information to YEH (i.e., using both online and offline methods) is an important factor to consider, given that not all YEH have regular access and use of technology, which may be further exacerbated during public health crises requiring physical distancing.

### Study Limitations

This scoping review has several limitations. We did not systematically assess the quality of studies given the heterogeneity of study methods, nor did we conduct a gray literature search. Similarly, due to time and human resources, only English language publications were included. It is therefore possible that some studies were omitted by the search strategy. In addition, as there was inconsistency in the ways in which papers reported their sociodemographic information and findings (e.g., eight papers reported on daily internet use, but only four of those papers additionally reported on weekly internet use), the weighted means reported in this review may not apply to the entirety of the study sample. Finally, as the present study is not a formal meta-analysis, we did not use more complex statistical pooling methods or analyze the heterogeneity in our data; as such, our results should be interpreted with these considerations in mind.

### Future Research

This scoping review highlights several research gaps, upon which we base the following recommendations: (1) international research is needed to understand YEH's access and use of ICTs, and to explore the impacts of varying infrastructures, government policies, and socioeconomic factors on YEH's experiences with technology; (2) more effort is needed to capture representative samples of the YEH population, characterizing the samples in terms of sociodemographic factors, and the role that these factors may play in their access and use of technology; (3) more consistency is needed in how access and use of technology is assessed and reported, as this will help to better synthesize the literature moving forward; (4) more research is needed on the digital health literacy skills of this population and their experiences of using technology to search for, and access health-related information and services; (5) quality appraisal will be an increasingly important consideration as more research emerges on access and use of ICTs among YEH; (6) more research is needed on how COVID-related public health guidelines affect access to publicly available ICTs (e.g., through libraries) and may further marginalize YEH from accessing critical health information and services; (7) future research should also focus on developing and evaluating technology-enabled health interventions for YEH. Indeed, we found that there is an emerging body of literature on the use of technology to deliver health related services to the homeless population, including youth. This is an important avenue to consider for a future review, to better examine the feasibility, acceptability, and efficacy of providing health services to YEH through this method of service delivery.

## Conclusion

Our findings indicate that YEH access and use ICTs for many purposes, and they appear to have the foundational skills, interests, and needs to engage with such types of technologies for health purposes. However, barriers to access need to be considered. More research is needed on the appropriate and effective way of leveraging ICTs for public health and health related interventions tailored for YEH. Given the urgency of YEH's health care needs and their marginalization from health care systems, it is important to pursue research on the impact of these technologies on YEH and health information and services for this vulnerable population.

## Data Availability Statement

The original studies presented in this review are included in the article/Supplementary Material, further inquiries can be directed to the corresponding author/s.

## Author Contributions

SL conceived the original idea for this scoping review, its overall methodology, and supervision of its implementation. SL and AF contributed to writing the initial protocol. SL and AH-A contributed to its revisions and finalization. SL prepared the initial draft of the manuscript, with contributions from AH-A. All authors contributed substantially to the content and approved the final version.

## Funding

SL is supported by a Canada Research Chair in Innovation and Technology for Youth Mental Health Services, and previously through a New Investigator Salary Award from the Canadian Institutes of Health Research.

## Conflict of Interest

SL has received an investigator-initiated, digital health operational research grant from Hoffman-La Roche, unrelated to this study. The remaining authors declare that the research was conducted in the absence of any commercial or financial relationships that could be construed as a potential conflict of interest.

## Publisher's Note

All claims expressed in this article are solely those of the authors and do not necessarily represent those of their affiliated organizations, or those of the publisher, the editors and the reviewers. Any product that may be evaluated in this article, or claim that may be made by its manufacturer, is not guaranteed or endorsed by the publisher.

## Abbreviations

YEH: Youth experiencing homelessness
ICTs: Information and communication technologies.
