# Supplementary material for: A Scoping Review on Access and Use of Technology in Youth Experiencing Homelessness: Implications for Healthcare
Source: Front Digit Health. 2021 Nov 24;3:782145. doi: 10.3389/fdgth.2021.782145 (PMC8651704; doi:10.3389/fdgth.2021.782145)
Supplement: Supplementary file 1 [file Table_1.pdf]

Appendix Table 1. Main objectives and results of studies reporting on access and use of ICTs by YEH.

Authors	Research Design	Methods	Sample N	% YEH	Age	Country	Objective	Results
Adkins et al., 2017	Observational, cross-sectional, descriptive	Focus group	24	100%	18-22 (M = 18.8, SD = 0.8)	USA	Examined the experiences and use of mobile phones and technology, health care needs and privacy concerns, and attitudes and interests of YEH towards technology and mental health services.	- All participants reported owning a mobile phone, which they used for communication (e.g., texting, phone calls), practical uses such as for navigation or as an alarm clock, entertainment (e.g., movies, music), and for social media.<br>- Participants reported that challenges maintaining the phone's functionality (e.g., finding ways to charge the phone) were a barrier to its use.
Barman-Adhikari & Rice, 2011[c]	Observational, cross-sectional, analytical	Survey	169	100%	13-24 (M = 20.9, SD = 2.1)	USA	Assess the percentage, characteristics, and social networks of YEH that use the internet to seek sexual health information, and the relationship between their social networks with their online sexual health information seeking.	- 54% reported using the internet at least once a day.<br>- Participants used the internet to connect with parents, home-based, and street-based peers, and to search for general and sexual health information, and health services.<br>- More youth with personal internet access (vs. youth with public internet access only) reported going online to seek

								health and sexual health information and services.
Barman-Adhikari et al., 2016	Observational, cross-sectional, analytical	Survey	1046 (only ran analyses on 829, as those that did not use social networking sites were excluded)	100%	13-25 (M = 21.34, SD = 2.16)	USA	Examine social networking site (SNS) use among YEH, specifically the extent of their use, content of their SNS conversations, and the recipients of their messages.	- 88% reported some form of social media use.<br>- 34% used social media daily, with 25% reporting weekly use.<br>- Participants used social media to communicate with peers, family members, and caseworkers, as well as for dating and relationship purposes.<br>- Connecting with street-based peers online, as well as discussing substance use and experiences of homelessness, was associated with an increased likelihood of engaging in concurrent sex and sex with someone met online.<br>- Discussing topics related to safe sex and goals, or connecting with a relationship partner online, was associated with positive sexual health behaviours, such

								as engaging in protected sex and getting a recent HIV test.
Curry et al., 2016	Observational, cross-sectional, analytical	Survey	642	100%	13-25 (M = 21.01, SD = 1.90)	USA	Understand the relationship between online and offline service use and the characteristics and behaviours of the YEH who use these services.	- 28% reported using the internet at least once a day, 45% at least once a week, and 27% less than weekly use.<br>- Participants used the internet for job-related activities (e.g., searching for a job), to search for housing, and to seek health services and information.<br>- Black participants and those living in shelters were more likely to use the internet to find housing.<br>- Those that became homeless due to mental health problems were more likely to use the internet to find health services.
Guadagno et al., 2013	Observational, cross-sectional, analytical	Survey	303	28%	Range unknown (M = 19.4, SD = 1.09)	USA	Examine the differences in YEH and housed undergraduates' use of communication technologies.	- 75% of YEH reported using social media.<br>- YEH used social media to communicate with peers and family, for dating and relationship purposes, and for educational and career networking.<br>- Compared to housed undergraduate students, YEH were more likely to send private messages to stay in touch with peers and relatives, and less likely to use

								social media to play online games.
Harpin et al., 2016	Observational, cross-sectional, analytical	Survey	181	100%	Range unknown (M = 20.61, SD = 0.151)	USA	Describe the characteristics of mobile phone ownership, telecommunications, and social media use among a sample of YEH.	- 47% owned a mobile phone, 65% of which being a smartphone.<br>- 72% regularly used social media.<br>- Participants reported accessing social media website through public Wi-Fi.<br>- Cell phones were purchased with personal money, with money specifically obtained through a job, obtained as a gift, or through another unspecified method.<br>- Participants who were able to find housing were more likely to regularly use social media (91%) than those living on the street (56%).
Hendry et al., 2017	Observational, cross-sectional, descriptive	Interview	36	53%	20-30 (M = N/A)	USA	Identify the barriers that YEH face when navigating the workforce, and how socio-technical systems can help them succeed and address these barriers.	- The following barriers were faced when using information-finding services to find job opportunities:<br>1. Individual barriers, such as lack of skills or training, requiring ID, etc.<br>2. Employer-related barriers, related to the values of potential employers<br>3. Societally-related barriers, such as a lack of available jobs<br>4. Technology-related barriers, such

								as needing to consistently access email when searching for a job.
Jennings et al., 2016	Observational, cross-sectional, descriptive	Focus group, survey	93 (Focus group n = 52; quantitative study group n = 41)	100%	Quantitative study: 15-24 (M = 18.1, SD = 0.4)<br><br>Qualitative study: 17-24 (M = 21.4, SD = not reported)	USA	Examine YEH' access to and use of mobile phones, as well as their preferences for mobile health interventions.	Quantitative findings (n = 41):<br>- 78% owned a mobile phone, 80% of which were smartphones.<br>- Cell phones were acquired as a gift, purchased with personal money or through federal benefits, or received through a free promotion.<br>Qualitative findings (n = 52):<br>- 90% owned a mobile phone.<br>- Participants often experienced episodes where their phones were disconnected, often due to financial constraints.
Karabanow & Naylor, 2010	Observational, cross-sectional, descriptive	Focus group, survey	20	100%	16-21 (M = N/A)	Canada	Explore how YEH understand, experience, and access information communication technology.	- The majority of participants used computers either daily or at least several times a week.<br>- Participants accessed computers either through a youth service drop-in centre or through a local public library.<br>- ICTs were used to communicate with peers and family, for general communication (e.g., email and instant messaging), to navigate social networking sites, to find housing, for job-

								related activities (e.g., resume development), and seeking general information online.<br>- A number of participants voiced their frustrations over the constraints of using public computers and lack of availability of devices.
Pollio et al., 2013	Observational, cross-sectional, descriptive	Survey	100	100%	18-24 (M = 20.4, SD = 1.8)	USA	Understand the circumstances (i.e., frequency, location, and purpose) surrounding YEH' use of technology, and the factors predicting their technology use.	- 46% accessed the internet at least once a day, with 93% reporting access at least once a week.<br>- Participants primarily accessed technology through social service agencies, libraries, internet cafes, through a friend or family member, or through a personal device.<br>- Their purposes for using technology were to communicate with peers or family, for general communication purposes, for work-related activities, for entertainment and leisure, for social networking, and for educational purposes.

Rice & Barman-Adhikari, 2014	Observational, cross-sectional, analytical	Survey	201 (only ran analyses on 194, as those that did not use the internet were excluded)	100%	13-24 (M = 21.06, SD = 2.05)	USA	Examine the social context in which YEH use social media, the extent of their use, and how it can connect them to positive relationships and online resource seeking.	- Participants reported that they had used the internet within the last two days (72%), on the same day (31%), the day before, or two days (10%) before responding to the survey.<br>- The internet was accessed through youth service agencies, public libraries, internet cafes, schools, work places, and through a personal or friend/family member's device.<br>- The internet was used for general communication (e.g., email), to navigate social networking sites, for entertainment and leisure, for job-related activities (e.g., job hunting), to find housing, and to communicate with peers, family members, and case workers.<br>- 57% reported using social media.<br>- Living situation (i.e., being literally homeless), time since first homelessness, and connecting to home-based peers, employers, and caseworkers online was associated with an increased likelihood of using the internet for job-related purposes.

Rice et al., 2010[b]	Observational, cross-sectional, analytical	Survey	201	100%	13-24 (M = 21.07, SD = 2.09)	USA	Examine the frequency of youth experience homelessness' online activity, their means of accessing the internet, the audience with which they are connecting, and the relationship between their technology use and health-related behaviours (i.e., engagement in exchange sex, HIV testing, and online partner-seeking).	- 30% reported accessing the internet at least once a day, with 55% reporting at least once weekly access, and 13% reporting less than weekly access.<br>- The internet was accessed through public libraries, youth service agencies, work places, internet cafes, at home or wherever they were staying, and through a personal or friend/family member's device.<br>- Participants used the internet to communicate with peers and family, to navigate social networking sites, and for dating and relationship purposes.<br>- Using the internet to communicate with street-based peers was associated with increased risky sex behavior, whereas communicating with family and home-based peers was associated with decreased risky sex behavior.
Rice et al., 2011a[c]	Observational, cross-sectional, analytical	Survey	169	100%	13-24 (M = 20.91, SD = 2.1)	USA	Examine the use and ownership of cell phones ownership among a sample of YEH, and its implications on their health behaviours.	- 62% reported owning a mobile phone.<br>- Mobile phones were obtained as a gift, purchased with money earned from a job or from pan-handling, or borrowed/shared with a friend.

								- Participants used mobile phones to communicate with peers, family, and caseworkers, and for job-related activities (e.g., communicating with potential or current employers).<br>- Identifying as LGBT+, living in a shelter or temporary housing, and older age were all associated with an increased likelihood of mobile phone ownership.
Rice et al., 2011b[a]	Observational, cross-sectional, analytical	Survey, interview	136	100%	13-24 (M = N/A)	USA	Examine the relationship between the composition of the social networks of YEH and substance use, with particular attention to the influence of home-based network ties via social networking technology.	- Participants reported using ICTs for general communication, and to communicate with peers, family, and caseworkers.<br>- Connections with home-based non-substance-using peers was associated with a reduction in substance use, whereas connecting with street-based substance-using peers was associated with an increase in substance use.
Rice et al., 2012[a]	Observational, cross-sectional, analytical	Survey, interview	136	100%	13-24 (M = 20.8, SD = 2.1)	USA	Examine the effects of social isolation on experiences of depression and anxiety in YEH, and how integration into street and home-based networks differentially	- Participants reported using ICTs to communicate with peers, and to navigate social-networking sites.<br>- Connecting with home-based peers through ICTs was associated with a reduction in depressive symptoms.

							impacts mental health outcomes.	
Robards et al., 2018	Observational, cross-sectional, descriptive	Interview	41	22%	12-24 (M = N/A)	Australia	Understand healthcare access, navigation of health care systems, and use of technology by marginalized youth.	- Participants reported using ICTs to seek health information and services and being more comfortable seeking help online.<br>- Google was a first portal of entry for seeking information, however participants varied in their ability to find information online.
VonHoltz et al., 2015	Observational, cross-sectional, analytical	Survey	67	100%	18-21 (M = 19.4, SD = 1.1)	USA	Understand the patterns of new-media use (both for general and health-related reasons) among YEH, and whether this use varies by their housing status.	- 87% reported accessing the internet at least once a day.<br>- Participants accessed the internet through a personal device or through a public computer.<br>- The internet was used to navigate social media sites, for job-related activities (e.g., job hunting), general communication (e.g., checking email), and to seek general and sexual health information and services.<br>- 85% of the sample reported using social media sites.<br>- Experiencing homelessness was

								associated with a decrease in internet use.
VonHoltz et al., 2018	Observational, cross-sectional, analytical	Survey, interview	87	100%	18-21 (M = 19.4, SD = 1.1)	USA	Examine characteristics of internet use (i.e., rates, duration of use, devices used to access) before and after living in a homeless situation, identify health-related behaviours engaged on the internet, and explore how technology can be used to provide resources to YEH.	- 56% reported accessing the internet at least once a day, with 86% reporting access at least once a week.<br>- The internet was accessed through a personal device, through a public computer, or through public Wi-Fi.<br>- Participants used the internet for job-related activities (e.g., job hunting), general communication (e.g., checking email), searching for housing, educational purposes (e.g., browsing school websites), to navigate social media websites, for entertainment and leisure, and to seek general and sexual health information and services.<br>- 85% of the sample reported using social media websites.<br>- Experiencing homelessness was associated with a decrease in internet access and a shift from using the internet for entertainment and leisure towards using it for goal-oriented activities (e.g., seeking housing and employment).

								- Smartphone ownership was associated with increased access to the internet.
Young & Rice, 2011[b]	Observational, cross-sectional, analytical	Survey	201	100%	13-24 (M = 21.07, SD = 2.09)	USA	Examine the relationship between online social networks, HIV risk behaviours, and knowledge about HIV and STI testing among YEH.	- 91% reported using social media, with 45% reporting daily use, and 34% reporting weekly use.<br>- Participants used social media to communicate with peers, and for dating and relationship purposes.<br>- Participants identifying as gay, lesbian, and bisexual were more likely to have used the internet for dating and relationship purposes.<br>- Participants who used social networks to discuss content related to drinking and drugs were more likely to engage in risky sex behaviours, whereas those who discussed love and safe sex were less likely to engage in risky sex behaviours.
a = Study findings are based on the same sample of YEH, recruited between June 19 and August 21, 2008<br>b= Study findings are based on the same sample of YEH, recruited between June 1 and June 22, 2009<br>c = Study findings are based on the same sample of YEH, recruited during the month of June, 2009 (no additional details provided)								

YEH: Youth experiencing homelessness; ICT: Internet communication technology; HIV: Human immunodeficiency virus; STI: Sexually transmitted infection
